# Double Blow in the Tropics: A Case of Concurrent Leptospirosis and Scrub Typhus

**DOI:** 10.7759/cureus.60732

**Published:** 2024-05-21

**Authors:** Nahas N Nazeera, Sreelakshmi M, Abin Mathew, Aneesh Basheer

**Affiliations:** 1 General Medicine, Dr. Moopen's Medical College, Wayanad, IND

**Keywords:** antibodies, thrombocytopenia, liver injury, typhus, leptospirosis

## Abstract

A 34-year-old male, with no history of known previous disease, employed at a ginger farm in South India, presented with a four-day history of high-grade fever and headache. Initially, he received symptomatic treatment but was referred due to hypotension and persistent fever. Investigations showed leucocytosis, thrombocytopenia, abnormal liver function tests, renal dysfunction, and elevated C-reactive protein. Positive results were obtained for *Leptospira* immunoglobulin M (IgM) and scrub typhus IgM tests, indicating a coinfection, reported rarely from this region. Timely clinical suspicion, prompt laboratory diagnosis, and early treatment with doxycycline and broad-spectrum antibiotics are crucial to prevent complications and fatal outcomes in such coinfections.

## Introduction

Leptospirosis and scrub typhus are two zoonotic diseases that pose significant health challenges in tropical and subtropical regions. Leptospirosis, caused by spirochetes of the genus *Leptospira*, is commonly transmitted through contact with water contaminated by the urine of infected animals like rats. Scrub typhus, on the other hand, is caused by *Orientia tsutsugamushi* and transmitted by the bite of infected chiggers. Both diseases present with a range of nonspecific symptoms, making diagnosis challenging and often leading to underreporting of co-infections [1,2]. Southern parts of India, bordering the Western Ghats, are an ideal niche for these two pathogens due to the geographic diversity, abundance of vegetation, availability of hosts, and recent increase in human-nature interactions due to tourism and urbanization. This creates unique opportunities for multiple pathogens to be transmitted from hosts through vectors to humans.

This report aims to shed light on the clinical presentation, diagnostic challenges, and management of a patient with concurrent leptospirosis and scrub typhus. The rarity of reports on such co-infections underscores the need for increased clinical awareness and availability of diagnostic tests, which are crucial for timely and effective treatment. By discussing this case, we hope to contribute to the growing body of knowledge on the management of these infections and highlight the importance of considering co-infections in patients with acute febrile illnesses in endemic regions [1,2]. The challenges associated with clinical and laboratory diagnosis of these two common infections in the tropics are discussed in this case report.

## Case presentation

A middle-aged gentleman, previously healthy with no known medical conditions, working at a ginger farm in South India, presented to the emergency department with a four-day history of high-grade fever and headache. He also complained of generalized body aches and fatigue. There was no history of rashes, cough, or yellowish discoloration of the eyes. He had no history of contact with persons suffering from fever and had not traveled out from his workplace recently. There was no history of dysuria, frequency of micturition, vomiting, or abdominal pain. 

Upon admission, the patient was conscious with a Glasgow Coma Scale (GCS) score of E4V5M6. The body temperature was 37.6°C, blood pressure was 70/60 mmHg, and the pulse rate was 90 beats per minute. The rest of the physical examinations were normal. Initial laboratory investigations revealed a white blood cell (WBC) count of 18,420/cu.mm, neutrophils of 97%, platelet count of 65,000/cu.mm, activated partial thromboplastin time (APTT) of 37.2 seconds, prothrombin time (PT) of 13.5 seconds, international normalized ratio (INR) of 0.98, and a highly positive C-reactive protein (CRP) level exceeding 300 mg/L. Electrolyte levels were normal. Renal function tests indicated a urea level of 77 mg/dL, serum creatinine of 3.2 mg/dL, and uric acid of 9.5 mg/dL. Liver function tests (LFT) showed total bilirubin (TB) of 1.96 mg/dL, direct bilirubin (DB) of 1.68 mg/dL, aspartate aminotransferase (SGOT) of 88 U/L, alanine aminotransferase (SGPT) of 60 U/L, and globulin of 3.7 g/dL. Urinalysis revealed a trace of sugar and 8-10 red blood cells (RBCs) per high power field. Initial blood gas analysis showed a pH of 7.36, partial pressure of carbon dioxide (PaCO_2_) of 32 mmHg, partial pressure of oxygen (PaO_2_) of 48 mmHg, bicarbonate (HCO_3_−) of 18.1 mmol/L, and an oxygen saturation of 96% on room air. Initial evaluations of dengue IgM and NS1 antigen were negative. Thick and thin smears for malarial parasites were also negative. With a provisional diagnosis of septic shock, the patient was commenced on a noradrenaline infusion.

**Figure 1:**
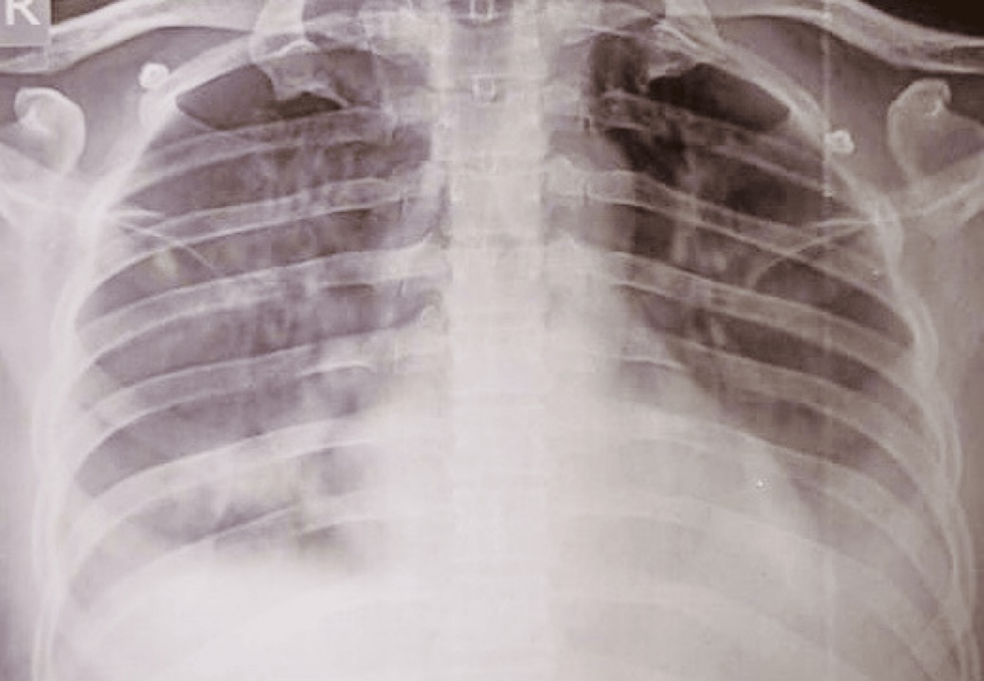
Chest X-ray of the patient demonstrating bilateral heterogenous infiltrates suggestive of acute respiratory distress syndrome (ARDS)

He was treated with intravenous meropenem (1 gm every eighth hour) to cover for possible septic shock, and tab doxycycline 100 mg twice daily orally was administered, considering tropical fever. Hydrocortisone 50 mg intravenously every sixth hour was also administered as an adjuvant therapy for refractory septic shock. Because of the declining urine output, the patient was started on diuretics. The patient's serum creatinine (from 3.2 to 6.8 mg/dl) and urea (from 71 to 181 mg/dl) values showed an increasing trend. There was a progressive worsening of thrombocytopenia also (Table 1). Haemodialysis was initiated given worsening urine output and progressively rising creatinine. A drop in saturation was noted (PaO_2_/FiO_2_ ratio 90), with a chest X-ray revealing bilateral pulmonary infiltrates, raising the probability of acute respiratory distress syndrome (ARDS) (Figure 1). Oxygen support was provided via nasal prongs and later through non-invasive ventilatory support due to hypoxia. Blood and urine cultures showed no growth. Scrub typhus immunoglobulin M (IgM) antibody and *Leptospira* IgM antibody (both by ELISA) done on day 7 of symptom onset returned positive. Antibiotics were de-escalated to ceftriaxone, while doxycycline was continued. With the initiation of hemodialysis, urine output improved and non-invasive ventilation (NIV) support was gradually tapered off as saturation levels improved, and the patient was shifted to the ward. During the stay, the patient's platelet count increased from 27,000 to 1.96 lakh/cu.mm, and creatinine values decreased from 6.6 to 2.4 mg/dl, with sustained adequate urine output. His general condition improved, and he maintained oxygen saturation on room air. He was discharged on the 14th day of hospitalization.

**Table 1:** Blood count and renal function trends during hospital stay

Parameter	Day 1	Day 2	Day 3	Day 4	Day 5	Day 6	Day 7	Day 9
Haemoglobin (g/dl)	16.1	14.3	13.2	12.7	14.7	13.6	13.9	12.1
Leucocyte count (cells/cu.mm)	18420	29500	15130	11660	8310	10950	12710	10000
Neutrophils (%)	97	96	89	79	76	80	72	75
Platelets (cells/cu.mm)	65,000	45,000	40,000	30,000	29,000	36,000	98,000	1,98,000
Blood urea (mg/dl)	77	80	119	166	166	171	131	95
Creatinine (mg/dl)	3.2	3.3	4.6	5.3	5.8	6.6	5.9	2.4

## Discussion

This gentleman displayed symptoms common to both leptospirosis and scrub typhus. Both infections present with fever, non-specific body aches, and involvement of multiple organs including the blood, liver, kidneys, and lungs. Our patient responded well to treatments including doxycycline, meropenem, and supportive care. 

Leptospirosis and scrub typhus are often overlooked tropical diseases with similar seasonal patterns. This creates a chance for both diseases to occur together. While cases of this coinfection have been reported in regions like Thailand [3] and Taiwan [4], there have been only a few such reports from India [2,5]. Leptospirosis tends to increase after the rainy season due to waterlogging and contact with animal urine, while scrub typhus rises with an increase in trombiculid mite populations in scrub growth. Therefore, primary physicians must consider the possibility of both diseases during the rainy season. Symptoms of both diseases can be nonspecific, including fever, headache, skin rash, and muscle pain. However, certain signs like calf tenderness and conjunctival suffusion point more toward leptospirosis, whereas an eschar suggests scrub typhus. In our patient, none of these signs were present, creating a diagnostic vacuum. In severe cases, both diseases can lead to various organ dysfunctions, making early diagnosis and treatment vital. Severe forms of both infections can cause acute liver injury, thrombocytopenia, and ARDS. Renal impairment, however, occurs more commonly in leptospirosis. One analytical study compared dengue-scrub coinfection versus isolated dengue from South India and identified several clinical and laboratory variables that could favor coinfection [6]. However, very few similar studies are available concerning leptospirosis-scrub coinfection. A recent study from Uttar Pradesh in India found a seroprevalence of leptospirosis-scrub co-infection in 8.4% of the samples tested from outpatients with short febrile illness [7]. However, this might not truly reflect the burden of co-infection considering that the study was based only on serological tests and did not include clinical criteria to define co-infection cases. Few studies have identified a higher median platelet count and a lower serum bilirubin and creatinine in co-infected patients compared to those with isolated leptospirosis [8].

The choice of antibiotics like doxycycline and meropenem, which cover both pathogens, is crucial for effective treatment. Starting with broad-spectrum antibiotics with appropriate coverage for common tropical infections using doxycycline and adjusting based on the patient's response is a sensible and widely accepted approach, particularly in highly endemic regions.

While cross-reactivity in ELISA testing for IgM antibodies can be an issue, clinical judgment based on symptoms should guide diagnostic decisions. If symptoms suggest both diseases, it is wise to test for both. It must also be noted that serological tests tend to overestimate the prevalence of co-infection and molecular testing using polymerase chain reaction (PCR) is ideal [9]. However, in individual patient settings, these tests may not always be available or affordable, and therefore a high index of suspicion along with positive antibody testing should be used judiciously for early detection and empiric treatment of such co-infections. This case emphasizes the importance of considering dual infections, like leptospirosis and scrub typhus, especially in regions where both are prevalent. Clinicians should be alert to the possibility of these infections, particularly in cases of undifferentiated acute febrile illness, alongside other common diseases like malaria, dengue fever, and typhoid fever. Early recognition and appropriate management can significantly improve patient outcomes.

## Conclusions

Despite the rarity of reported coinfections from South India, it is important to raise clinical awareness and improve diagnostic test availability. Although the clinical picture of leptospirosis and scrub typhus overlap, very severe manifestations including sepsis, septic shock, and ARDS during rainy seasons in endemic areas like ours must alert clinicians to the possibility of co-infection. It is worthwhile to test for both infections in such patients and empirically cover for both organisms until definitive test results are available. Early detection and appropriate treatment can prevent complications and fatal outcomes. Our case highlights the need for a high index of suspicion for co-infections in endemic areas and systematic research into the epidemiology, risk factors, and true burden of co-infections in South India.
